# Classical autophagy-modulating compounds impair viability and alter autophagy gene expression in schistosomula and adult *Schistosoma mansoni*

**DOI:** 10.3389/fpara.2026.1892940

**Published:** 2026-10-27

**Authors:** Agatha Fischer-Carvalho, Larissa Carolina Lopes-Jesus, Sergio Verjovski-Almeida, Murilo Sena Amaral

**Affiliations:** 1 Laboratório de Ciclo Celular, Instituto Butantan, São Paulo, Brazil; 2 Programa de Pós-Graduação Interunidades em Biotecnologia, Universidade de São Paulo, São Paulo, Brazil; 3 Departamento de Bioquímica, Instituto de Química, Universidade de São Paulo, São Paulo, Brazil

**Keywords:** autophagy, chloroquine, gene expression, rapamycin, *Schistosoma mansoni*, spautin-1, *TOR*

## Abstract

**Introduction:**

Schistosomiasis is a neglected disease caused by blood flukes affecting millions worldwide and leading to substantial morbidity and economic losses. Despite its global impact, treatment still relies almost exclusively on praziquantel (PZQ); however, the possible emergence of *Schistosoma* strains with reduced sensitivity to PZQ, alongside PZQ limited efficacy against pre-patent infections, highlights the need for new multi-stage therapeutic approaches. In this context, classical autophagy-modulating compounds may represent valuable tools for elucidating *Schistosoma mansoni* biology.

**Methods:**

The effect of rapamycin, a classical autophagy inducer, was evaluated in *S. mansoni* schistosomula through total ATP quantification and PI/FDA assays. In adult worms, besides ATP quantification, additional phenotypic evaluations included worm pairing, adhesion to culture plates, motility, and egg production. Expression levels of selected autophagy genes were measured by RT-qPCR in both developmental stages. In addition, compounds commonly used to inhibit autophagy in other organisms (bafilomycin A1, chloroquine, spautin-1, and wortmannin) were tested in schistosomula using ATP quantification, and their effects on autophagy genes were evaluated by RT-qPCR in schistosomula and adult worms.

**Results:**

Rapamycin reduced schistosomula and adult worm viability, presenting with an IC_50_ of 14.9 μM and 4.6 μM, respectively, and modulated the expression of several autophagy genes in both parasite stages. Notably, a putative *TOR* orthologue (*Smp_122910.1*) was downregulated in male and female worms exposed to a sublethal dose of rapamycin. Although schistosomula ATP levels were not altered by the four inhibitors tested, chloroquine modulated autophagy gene expression.

**Discussion:**

These findings reveal the antischistosomal potential of rapamycin and identify autophagy genes altered across developmental stages, including *Lysosome-associated membrane glycoprotein* (*Smp_073400.1*) and *Phospholipase B-like* (*Smp_301430.1*). Stage-specific modulation of the putative *TOR* orthologue suggests a drug-responsive gene candidate for further functional assays, positioning the tested compounds as promising pharmacological tools for investigating schistosomes biology.

## 1 Introduction

Schistosomiasis is a neglected disease caused by blood flukes from the genus *Schistosoma*, whose transmission has been reported in 79 countries (WHO- World Health Organization, 2026). The disease is considered the second most important parasitic infection (CDC- U. S. Centers for Disease Control and Prevention, 2024), with prevalence reaching up to 23.2% in endemic regions (Temesgen et al., 2026), and causes alarming socioeconomic impacts, achieving 1.7 million disability adjusted life-years (DALYs) in 2021 (Li et al., 2025). The human-infective larvae (cercariae) are released from the snail host into freshwater and penetrate the host’s skin, becoming juvenile larvae (schistosomula) that fully develop into adult worms upon reaching the hepatic portal vein (Colley et al., 2014). Despite the epidemiological relevance of the disease, praziquantel (PZQ) has been the only available drug for treatment. Importantly, reports of *S. mansoni* strains with low sensitivity towards PZQ (Ismail et al., 1996; Melman et al., 2009), and the reduced efficacy of PZQ against immature stages, limiting its effectiveness during pre-patent infections (Xiao et al., 1985; Pica-Mattoccia and Cioli, 2004), underscore the need for alternative therapeutic strategies to tackle schistosomiasis. In this context, deepening the understanding of schistosomes biology is crucial for identifying new therapeutic targets against these parasites.

Macroautophagy (hereafter referred to as autophagy) is a highly conserved pathway crucial for maintaining cellular homeostasis (Levine and Kroemer, 2019) and cytoprotective stress management (Kroemer et al., 2010), primarily through the recycling of damaged or dispensable cellular components. Although it occurs constitutively at basal levels, autophagy is modulated under adverse conditions, acting as a key regulatory mechanism in diverse biological contexts, including cancer (Jin and White, 2007; Huang et al., 2016) and parasitic infections (Cervantes et al., 2014). In the context of infectious diseases, autophagy has been mainly investigated as a mediator of host response (Ghartey-Kwansah et al., 2020; Rashidi et al., 2021; Silva et al., 2024) and as an immune evasion strategy exploited by pathogens (Zhou et al., 2022; Shariq et al., 2023), with the autophagy machinery of pathogens being progressively highlighted as a potential therapeutic target. In schistosomes, the autophagy machinery components and their putative roles in homeostasis or response to stress conditions remain poorly understood.

In *S. mansoni*, autophagy has been little explored, with existing research primarily limited to exposing the parasite to conditions known to modulate autophagy in other organisms. These include *in vitro* culture with rapamycin, a potent inducer of autophagy, with two reports on its effect on adult *S. mansoni* parasites (Rossi et al., 2002; Mughal et al., 2021): in 2002 (Rossi et al., 2002) reported that adult *S. mansoni* worms were insensitive to rapamycin *in vitro* exposure at 22 and 44 μM, while (Mughal et al., 2021) subsequently have shown that rapamycin at concentrations as low as 5 μM modulated autophagy in adult *S. mansoni* worms, as shown by an increased LC3B-II: LC3B-I ratio. Canonical autophagy inhibitors such as lucanthone (Clarkson and Erasmus, 1984), bafilomycin A1, spautin-1, wortmannin (Mughal et al., 2021), and chloroquine (Keiser et al., 2014) have also been tested in adult worms. In addition, compounds shown to modulate autophagy in schistosomes as part of their mechanism of action have also been explored, such as metformin (Abdel Menaem et al., 2025) and imatinib (Beckmann and Grevelding, 2010; Mughal et al., 2022). However, how classical compounds known to modulate autophagy in other organisms affect *S. mansoni* schistosomula viability and gene expression remains unexplored.

Since several autophagy modulators are clinically approved or commercially available, they have attracted considerable attention as repositioning candidates (Bu et al., 2022; Kench et al., 2024), which could also be explored as putative pharmacological tools against helminths. To date, the effects of rapamycin and other drugs generally used to disrupt autophagy have not been tested on schistosomula, a life-cycle stage that most certainly involves autophagy as the schistosomula undergoes extensive cell and tissue reprogramming towards development into adult parasites (Crabtree and Wilson, 1986). In this respect, in 3-day-old schistosomula that were killed by the *in vitro* exposure for an additional two days to plasma from infected and self-cured rhesus macaques, a significant decrease has been described in the expression of genes within the autophagy machinery (Amaral et al., 2021). Thus, despite the growing evidence of autophagy’s key role in Apicomplexa parasites such as *Plasmodium* and *Toxoplasma* (Besteiro, 2017; Sakamoto et al., 2021), the extent to which the exposure to canonical autophagy modulators may affect *Schistosoma* survival across different life-cycle stages remains poorly understood.

In this study, we investigated the effect of compounds classically used to perturb autophagy on *S. mansoni* viability, contributing to the understanding of the responsiveness of the parasite to these pharmacological perturbations. Rapamycin, a canonical inducer of autophagy, was evaluated against schistosomula and adult worms, while four compounds known to inhibit autophagy in other organisms (bafilomycin A1, chloroquine, spautin-1, and wortmannin) were screened against schistosomula. Moreover, we have evaluated autophagy genes whose expression levels are responsive to rapamycin and chloroquine in both parasite stages. Altogether, these findings advance our understanding of the transcriptional pattern of *S. mansoni* following pharmacological treatment and underscore these compounds’ potential as a promising pharmacological strategy for investigating schistosomes biology.

## 2 Materials and methods

### 2.1 Parasite material

All experiments were performed using the BH (Belo Horizonte) strain of *S. mansoni*. The parasite life-cycle was maintained through cyclical passages in golden hamsters (*Mesocricetus auratus*) and *Biomphalaria glabrata* snails. Cercariae were obtained from snails 30 days post-infection (dpi), following a 2-h light exposure to induce shedding. Newly transformed schistosomula (NTS) were obtained by mechanical transformation of cercariae and separation of their bodies as previously described in (Basch, 1981), with some modifications. Briefly, cercariae were collected as described above and then suspended in 15 ml of M169 medium (Vitrocell) containing 2% penicillin/streptomycin, 25 μg/ml amphotericin B (Nova Biotecnologia), 0.5% gentamicin (Gibco, Life technologies) and 120 μM 5-fluorocytosine (Sigma-Aldrich). Mechanical transformation was performed by passing the cercariae 13 times through a 23G needle followed by ten resuspensions in 6 ml of M169 supplemented medium, to separate the decanted schistosomula from the tail-rich supernatant, as described in (Amaral et al., 2020). The NTS were maintained for 72 h in M169 medium supplemented with 2% penicillin/streptomycin, 25 μg/ml amphotericin B, 0.5% gentamicin, 120 μM 5-fluorocytosine, 2% fetal bovine serum, 1 μM serotonin, 0.5 μM hypoxanthine, 1 μM hydrocortisone, and 0.2 μM triiodothyronine at 37 °C and 5% CO_2_. For schistosomula, each biological replicate consisted of a pool of cercariae recovered from approximately 30 different snails, previously infected at distinct dates with different cercariae, thus representing independent cercarial transformations.

For adult *S. mansoni* obtention, golden hamsters were infected with 250 cercariae and submitted to portal perfusion using RPMI-1640 medium (Gibco, Life technologies) on the 49th dpi. After perfusion, adult worm couples were kept for 3 h at 37 °C and 5% CO_2_ in ABC169 media (Wang et al., 2019; Silveira et al., 2023) until incubation with the tested compounds. For adult worms, each biological replicate consisted of a pool of worms recovered from five hamsters previously infected at different dates with different cercariae shed from different snails.

### 2.2 Parasite incubation with classical autophagy modulators and IC_50_ determination

Rapamycin (Sigma-Aldrich, CAS 53123-88-9), chloroquine (Sigma-Aldrich, CAS 50-63-5), spautin-1 (Sigma-Aldrich, CAS 1262888-28-7), and wortmannin (Sigma-Aldrich, CAS 19545-26-7) were dissolved in DMSO to stock concentrations of 10 to 50 mM. Bafilomycin A1 (Sigma-Aldrich, CAS 88899-55-2) was obtained as a ready-to-use solution. All compounds were diluted in culture medium to the desired final concentrations. Vehicle control groups were cultured in a culture medium with a volume of DMSO equivalent to that used in the highest concentration of the compound tested in each experiment.

For determination of the half maximal inhibitory concentration (IC_50_) in schistosomula, one hundred schistosomula that had been previously cultured *in vitro* for 3 days were incubated *in vitro* per well in a 96-well plate for up to 120 h in 100 μl of supplemented M169 medium, containing rapamycin (0.1 – 50 μM), bafilomycin A1 (0.005 – 2.5 μM), chloroquine (0.4 – 200 μM), spautin-1 (0.4 – 400 μM), or wortmannin (0.2 – 100 μM) at serial two-fold dilutions, without drug replenishment. DMSO (12% v/v) and auranofin (10 μM) were used as negative and positive controls, respectively. For gene expression analysis, four to ten thousand schistosomula previously cultured *in vitro* for 3 days were incubated for an additional 48 h in supplemented M169 medium with 10 μM rapamycin or 100 μM chloroquine, with replenishment of chloroquine every 24 h. DMSO (0.2% v/v) was used as negative control.

Since the effects of bafilomycin A1, spautin-1, wortmannin, and chloroquine were previously determined in *S. mansoni* adult worms (Mughal et al., 2021; Keiser et al., 2014), we determined IC_50_ for rapamycin in adult worms. For IC_50_ determination, five worm couples per biological replicate (BR) were incubated *in vitro* in 1 ml of ABC169 medium containing rapamycin at serial concentrations (0.4 – 50 μM, at 2-fold dilution increments) for 48 h, with drug replenishment every 24 h. As negative control, DMSO (0.5% v/v) was used.

For gene expression analysis, five worm couples were incubated *in vitro* in 2 ml of ABC169 medium per well with either 5 μM rapamycin, or 50 μM spautin-1, or 200 μM chloroquine for 48 h, with drug replenishment every 24 h. As negative control, DMSO (1% v/v) was used. Three to four BRs were performed, and five to ten worm couples were used in each condition for each BR.

### 2.3 Parasite viability evaluation and phenotypic analysis

#### 2.3.1 Schistosomula

Parasite viability following exposure to the tested compounds was determined by a cytotoxicity assay (CellTiter-Glo Luminescent Cell Viability Assay, G7570, Promega), which quantifies ATP levels as an indicator of metabolically active cells. For schistosomula viability assessment, 100 μl of CellTiter-Glo reagent was added directly to the wells containing one hundred schistosomula each, followed by incubation for 5 min under agitation, and luminescence read using a Varioskan LUX Microplate Reader (Thermo Fisher Scientific). For the time curve, an independent 96-well plate was used per biological replicate, with each well being used for ATP quantification only for one specific time point; each experimental condition (concentration) was evaluated by 2 technical replicates per concentration per plate, across 4 biological replicates. Schistosomula morphology and motility were recorded every 24 h using an Olympus CKX41 inverted microscope at 10× magnification, and images were acquired by a digital camera (SC30, Olympus, Germany) with CellSens Dimension software (Olympus). **Supplementary Videos 1–8** are provided in **Supplementary Material**.

Schistosomula viability was also evaluated by propidium iodide (PI, 2.0 µg/ml; Sigma-Aldrich) and fluorescein diacetate (FDA, 0.5 µg/ml; Sigma-Aldrich) staining, as described in (Peak et al., 2010; Amaral et al., 2021), using auranofin at 10 μM as positive control. Schistosomula were observed by light microscopy at 5× magnification using a microscope Leica DM IL LED, with dead schistosomula recorded by a red fluorescence signal (572 nm emission filter) and live schistosomula by a green signal (492 nm emission filter). Raw count of live and dead schistosomula in each condition are provided in **Supplementary Table S1**. Percentage of FDA-positive schistosomula were calculated compared with the control group (DMSO at 0.25% v/v). Experiments were performed with three BRs, each containing two technical replicates.

#### 2.3.2 Adult worms

For adult worm viability assessment, control or treated worm couples were collected from the plate, washed in PBS, lysed by trituration and 100 μl of CellTiter-Glo reagent was added to the freshly lysed parasites. Luminescence was read as described for schistosomula. Experiments were performed with three to four BRs, each containing two technical replicates.

Adult worm phenotypic evaluations were assessed every 24 h by optical microscopy (Olympus CKX41). Three parameters were evaluated: pairing status, adhesion to the plate, and motility. Worm motility was scored according to (Horiuchi et al., 2005), which defines three motility scores: score 3 (full-body movement), score 1.5 (partial movement or live but immobile worms), and score 0 (worms considered dead). The motility index and relative motility index were calculated as described in (Horiuchi et al., 2005). For egg quantification, the medium in which parasites were cultured was collected, with subsequent PBS washing to ensure complete collection of eggs. The medium was briefly centrifuged for egg deposition, the volume was adjusted to 1 ml and 100 μl were taken for egg counting estimation under a bright-light 4× magnification using an Olympus CKX41 inverted microscope. The IC_50_ values and viability impacts of the tested compounds were calculated from pairing, adhesion, motility, egg count, and ATP levels of *S. mansoni* couples using GraphPad Prism v.8.4.3 (GraphPad Software).

### 2.4 RNA extraction, quantification, and quality assessment

For schistosomula, four to ten thousand parasites were washed in PBS after incubation with the compounds, centrifuged and resuspended in an RLT buffer (Qiagen) supplemented with 2-mercaptoethanol. Total RNA was extracted using the RNeasy Plus Micro Kit (Qiagen), as described in (Amaral et al., 2021). For adult worm RNA extraction, ten adult males and ten adult females from each condition were washed in PBS, then stored in RNAlater (Ambion) at 4 °C for 24 h, followed by storage at −80 °C. After thawing, worm couples were manually separated using tweezers under a stereomicroscope and total RNA extraction was separately performed from males or females, as described for schistosomula above. The integrity of all RNAs was verified using the Agilent RNA 6000 Pico (5067-1513, Agilent Technologies) or Nano kit (5067-1511, Agilent Technologies) in a 2100 Bioanalyzer Instrument (Agilent Technologies). Samples were quantified using the Qubit RNA HS Assay Kit (Q32852, Thermo Fisher Scientific). Purity was assessed by 260/280 nm and 260/230 nm ratios using Nanodrop (Thermo Fisher Scientific). Three to four BRs were assessed for adult worms, while three to five BRs were assessed for schistosomula.

### 2.5 Primer design

All primers were supplied by Thermo Fisher Scientific, and were either retrieved from literature (Mughal et al., 2021; Silveira et al., 2021) or designed using the Primer3Plus software tool (https://www.primer3plus.com/index.html, accessed on 10 October 2023), following the parameters described in (Jardim Poli et al., 2024). Primer efficiency was assessed by standard curve using 2-fold serial dilutions (1:8 steps) of pooled *S. mansoni* male and female complementary DNA (cDNA) samples, with RT-qPCR performed as described in section 2.6. All primer sequences are provided in **Supplementary Table S2**.

### 2.6 Reverse transcription and quantitative PCR assays

Reverse transcription (RT) reactions were performed using SuperScript IV First-Strand Synthesis System (18091050, Life Technologies), using 50 ng/µl of random hexamers. For schistosomula samples, RT reactions were performed using 25 ng of total RNA, and the resulting cDNAs were diluted 4× in DEPC-treated water. For adult worm samples, 140 ng of total RNA from each sample were used for RT reactions, and cDNAs were diluted 10× in DEPC-treated water. Quantitative PCR (qPCR) was performed using 5 µl of 1× LightCycler 480 SYBR Green I Master Mix (04707516001, Roche Diagnostics), 2.5 µl of cDNA samples, and 800 nM of each primer on a LightCycler 480 System (Roche Diagnostics). Each reaction was run in three technical replicates, in addition to the no template control (NTC). Results were analyzed using the comparative Ct method (Livak and Schmittgen, 2001), with primer efficiency correction. Raw Ct values are provided in **Supplementary Tables S3**–**S5**. All RT-qPCR data were normalized to the expression levels of reference genes found as the most stable in each condition according to the RefFinder tool (https://www.ciidirsinaloa.com.mx/RefFinder-master/, accessed on 17 March 2026). For schistosomula, *Smp_041060.1* and *Smp_062630.1* were selected by RefFinder as the most stable genes and used as normalizers. For adult worms, previously validated reference genes retrieved from (Silveira et al., 2021) were used. The results of the selection of reference genes by RefFinder are provided in **Supplementary Table S6**. Fold changes were calculated by dividing the treatment group gene expression value by the control group gene expression value.

### 2.7 Gene expression patterns of selected autophagy genes across *S. mansoni* life-cycle stages

Plots showcasing the expression patterns for the selected protein-coding genes across *S. mansoni* life-cycle stages (miracidium, sporocyst, cercariae, schistosomula, adult male, and adult female) were generated using publicly available RNA-Seq normalized counts (TPM, transcript per million) from (Silveira et al., 2021), available at https://verjolab.shinyapps.io/Reference-genes/ (accessed on 23 March 2026).

### 2.8 Single-cell clusters search of autophagy genes

The *S. mansoni* single-cell clusters atlas publicly available at *Schistosoma mansoni* Database (https://schisto.xyz/, accessed on 22 March 2026) (Diaz Soria et al., 2020; Wendt et al., 2020) for schistosomula and adult worms were used to identify the cell type in which each autophagy gene tested by RT-qPCR is expressed and significantly enriched.

### 2.9 Statistical analyses

Statistical analyses were performed using GraphPad Prism v.8.4.3. Schistosomula viability was analyzed by two-way ANOVA. For adult worms, two-way ANOVA was used to analyze worm pairing, adhesion, and motility, while one-way ANOVA was used to assess oviposition and percentage of ATP in each condition. All ANOVA analyses were performed using Dunnett’s *post-hoc* correction. Dose-response curves and IC_50_ values were estimated by fitting a five-parameter logistic (5-PL) model. Differences in relative gene expression between treatment and DMSO control groups, as determined by RT-qPCR, were evaluated using unpaired two-tailed Student’s *t*-test. Student’s *t*-tests were additionally applied to compare the culture medium and vehicle (DMSO) controls as an internal quality check. Values of p *<* 0.05 were considered statistically significant. Data are representative of the mean ± SEM of three to five independent experiments.

## 3 Results

### 3.1 Rapamycin *in vitro* incubation decreases *S. mansoni* schistosomula viability

To evaluate the antischistosomal activity of rapamycin against juvenile *S. mansoni*, 3-day-old schistosomula were exposed *in vitro* to rapamycin at concentrations ranging from 0.1 to 50 μM for up to 120 h, with viability assessed daily by ATP quantification. After 72 h of exposure to concentrations above 25 μM rapamycin, an almost complete (> 98%) loss in schistosomula viability was noticed (**Figure 1A**). The IC_50_ of rapamycin against *S. mansoni* schistosomula, determined from the ATP-based dose-response curve following a 48-h incubation, was 14.9 μM (**Figure 1B**). Incubation for 48 h with rapamycin at 25 μM confirmed schistosomula death, leading to a 69.6% mortality rate, assessed by PI and FDA staining (**Figures 1C, D**). Incubation with rapamycin induced morphological changes in schistosomula compared with control parasites, such as a dark, swollen, and granular appearance (**Supplementary Figure S1**), with some of them presenting large vacuoles. At concentrations above 25 μM, a complete absence of movement was observed, in contrast to control schistosomula (**Supplementary Videos 1–3**).

**Figure 1:**
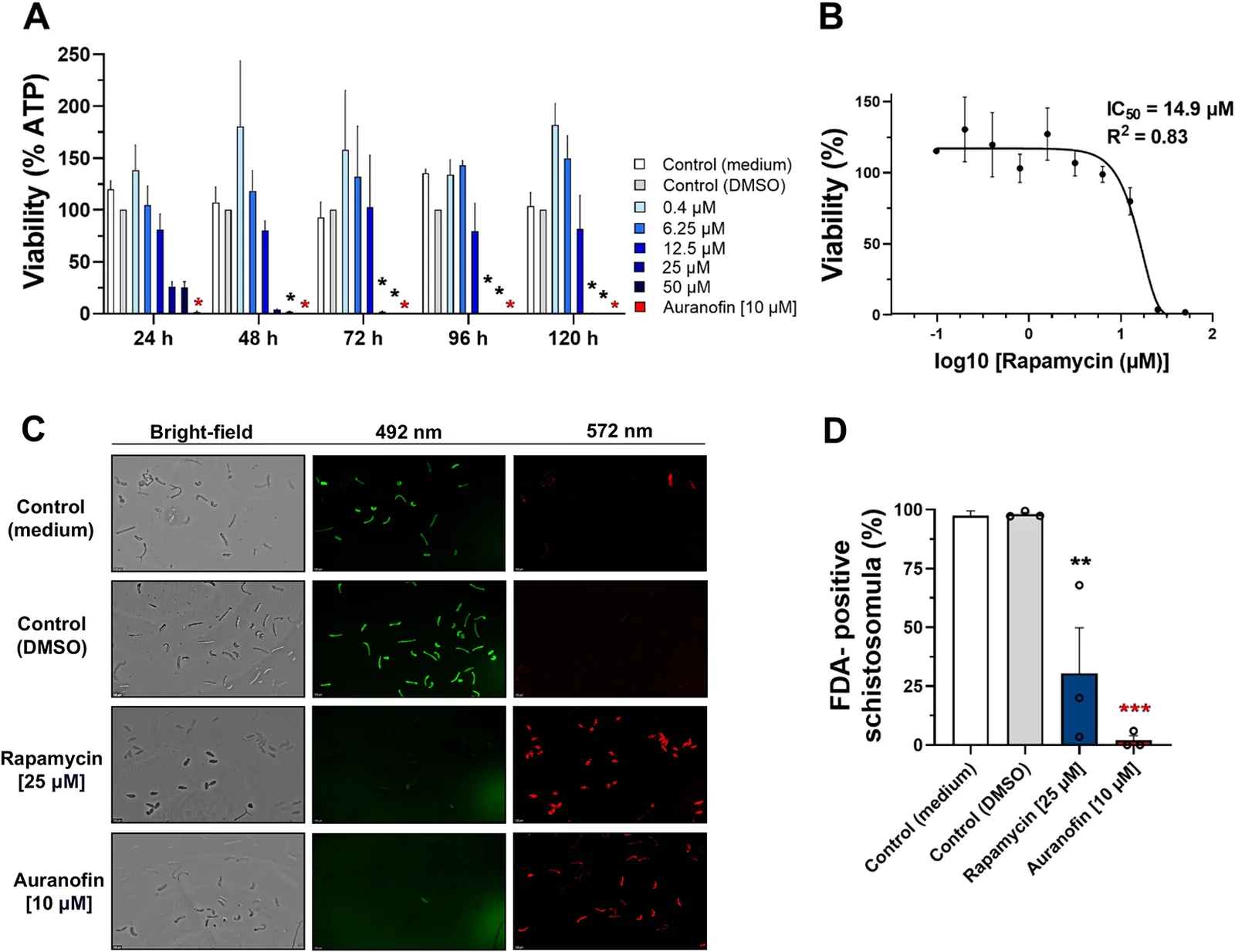
Evaluation of *Schistosoma mansoni* schistosomula viability following *in vitro* incubation with rapamycin. **(A)** 3-day-old schistosomula were incubated *in vitro* with rapamycin at concentrations ranging from 0.1 to 50 μM and the effect on parasite viability was observed for up to 120 h following rapamycin addition; five representative rapamycin concentrations are shown. Negative controls represent schistosomula incubated with medium or DMSO. Viability of schistosomula is shown as % ATP relative to the control following incubation with several concentrations of rapamycin for up to 120 h, with statistically significant differences determined by two-way ANOVA with Dunnett’s *post-hoc* correction. **(B)** Dose-response curve and calculated IC_50_ for schistosomula viability based on ATP quantification following incubation with rapamycin for 48 h, fitted using a five-parameter logistic (5-PL) model. **(C)** Fluorescence-based assessment of parasite viability following a 48-h incubation with 25 μM rapamycin. Images represent the same field of view acquired by bright-field, fluorescence at 492 nm (FDA channel), and fluorescence at 572 nm (PI channel), respectively. Viable parasites stained with fluorescein diacetate (FDA) emit green fluorescence (492 nm), whereas non-viable parasites stained with propidium iodide (PI) emit red fluorescence (572 nm). Auranofin at 10 μM was used as positive control. **(D)** Proportion of live schistosomula (expressed as % FDA-positive schistosomula) assessed by propidium iodide (PI) and fluorescein diacetate (FDA) staining following a 48-h incubation with rapamycin at 25 μM, compared with the DMSO control; unpaired two-tailed Student’s *t*-test. Auranofin at 10 μM was used as a positive killing control. Bar at the bottom left: 100 μm. Significance: (*) = p <0.05, (**) = p <0.01, (***) = p <0.001. Values are expressed as the mean ± SEM of three to four independent biological replicates.

### 3.2 Rapamycin *in vitro* incubation triggers modulation of expression levels of autophagy genes in *S. mansoni* schistosomula

To investigate a possible modulation of the expression of autophagy genes induced by rapamycin, we selected five genes putatively associated with autophagy for quantification using RT-qPCR, namely *Lysosome-associated membrane glycoprotein* (*Smp_073400.1*), *Phospholipase B-like* (*Smp_301430.1*), putative *TOR* orthologue gene (*Smp_122910.1*), and two putative *LC3B* genes (*Smp_073790.1* and *Smp_087940.2*). Schistosomula incubated *in vitro* with rapamycin at 10 μM for 48 h showed a marginally significant 4-fold downregulation in the expression levels of *Smp_073400.1* (**Figure 2A****),** and a significant 4-fold downregulation of *Smp_301430.1* (**Figure 2B**) compared with the control DMSO group. However, no significant changes in the expression levels were detected for the putative orthologue genes encoding *TOR* (*Smp_122910.1*) or *LC3B* (*Smp_073790.1* and *Smp_087940.2*) (**Figures 2C–E**).

**Figure 2:**
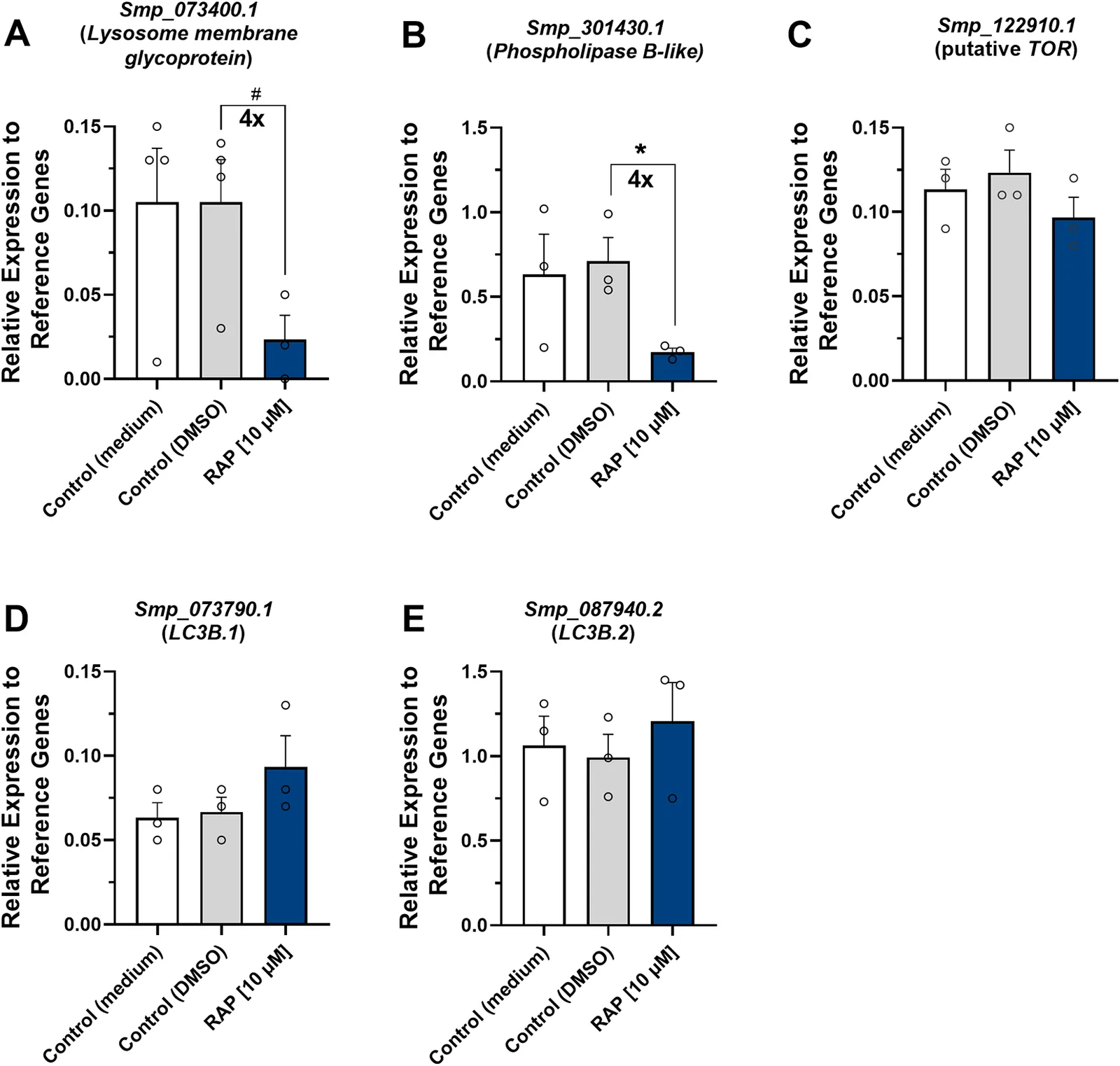
Effect of rapamycin on the expression levels of autophagy genes in *Schistosoma mansoni* schistosomula. Relative transcript levels of **(A)** *Lysosome membrane glycoprotein* (*Smp_073400.1*), **(B)** *Phospholipase B*-*like* (*Smp_301430.1)*, **(C)** putative *TOR* (*Smp_122910.1*), **(D)** *LC3B.1* (*Smp_073790.1*), and **(E)** *LC3B.2* (*Smp_087940.2*) were determined by RT-qPCR following *in vitro* incubation with rapamycin at 10 μM for 48 h. The relative expression levels were calculated using the delta Ct approach considering each pair of primers efficiency and normalized to the geometric average of two selected reference genes (*Smp_062630.1* and *Smp_041060.1*). Statistically significant differences between the treatment and DMSO control groups were assessed using a two-tailed Student’s *t*-test, with the number below the brackets indicating the fold-changes (FCs). Significance: (#) = p <0.053, (*) = p <0.05. Values are expressed as the mean ± SEM of three to four independent biological replicates. RAP, Rapamycin.

### 3.3 Rapamycin *in vitro* incubation impairs adult *S. mansoni* pairing, adhesion, motility, oviposition, and ATP levels

Given rapamycin’s robust phenotypic effects against schistosomula (**Figure 1A**) and its known capacity to modulate autophagy in adult *S. mansoni* (Mughal et al., 2021), we investigated its impact on adult worms’ phenotypic parameters following *in vitro* incubation with a range of concentrations of rapamycin (from 0.4 to 50 μM) for up to 48 h. Pairing stability, adhesion to the plate, and motility were monitored daily, while egg production and viability (% ATP) were assessed 48 h after incubation with rapamycin. For all parameters, a dose dependent effect was noticed (**Figures 3A–E**). At 12.5 μM concentration, couples pairing and adhesion to the plate were severely impaired after 24 h of incubation, showing a 100% (**Figure 3A**) and 80% (**Figure 3B**) decrease, respectively. After 48 h, a complete impairment in pairing, adhesion to the plate, and motility was noticed following incubation with rapamycin at a concentration as low as 12.5 μM (**Figures 3A–C**). Interestingly, motility impairment at 24 h was less pronounced than the effects observed on pairing and adhesion, as significant reductions were only detected at the two highest concentrations tested (25 and 50 μM) (**Figure 3C**). These findings suggest a temporal progression of rapamycin-induced effects, in which impairment of worm pairing and plate adhesion precedes motility reduction, the latter becoming most pronounced after 48 h of incubation.

**Figure 3:**
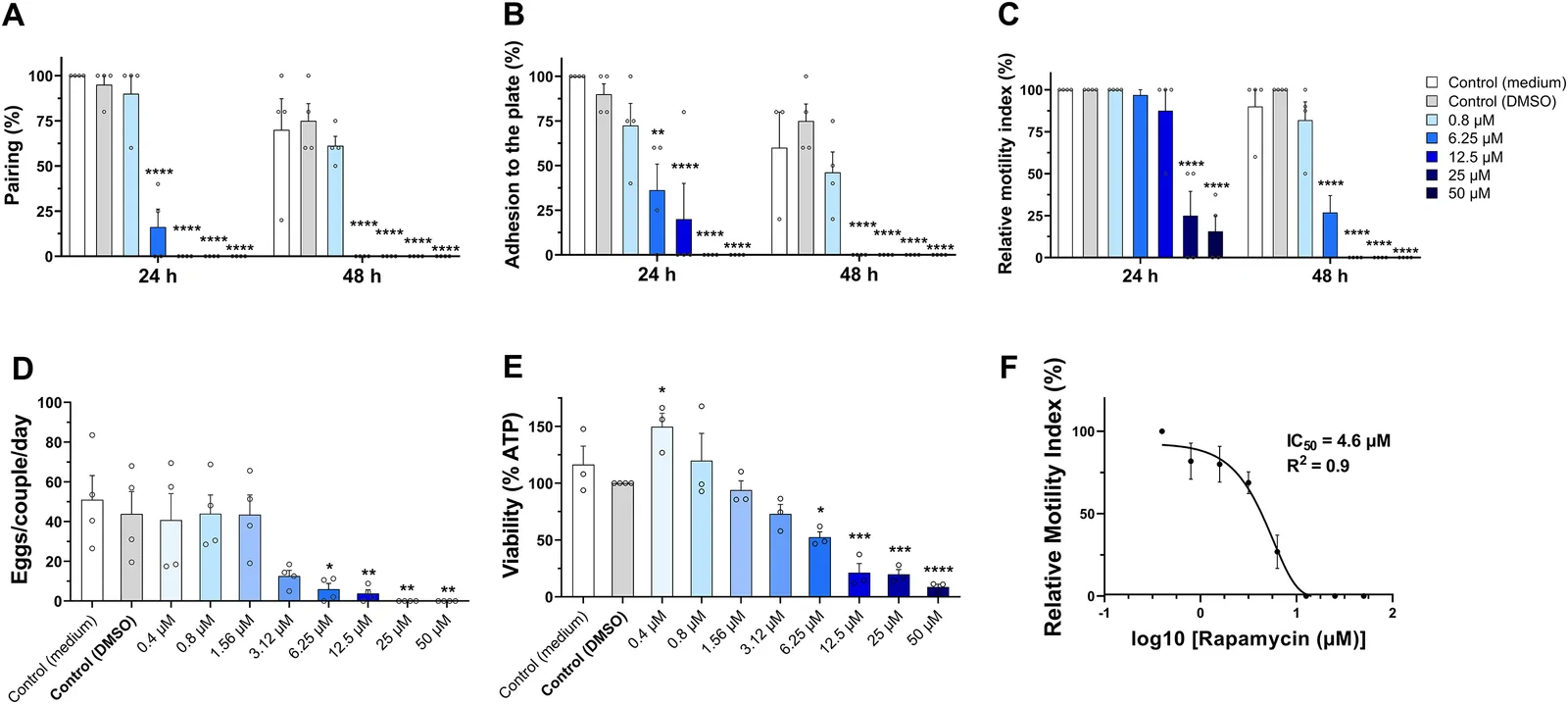
Effect of rapamycin on adult *Schistosoma mansoni* worms’ viability. Adult worms were incubated *in vitro* for 24 or 48 h with rapamycin at concentrations of 0.4 to 50 μM; five representative concentrations are shown **(A–C)**. As a negative control, DMSO was used. The effects on the following viability parameters were recorded: **(A)** Percentage (%) of paired worms. **(B)** Percentage (%) of couples adhered to the plate. **(C)** Relative motility index (% compared with the DMSO control group) of worm couples. **(D)** Egg quantification per couple per day. **(E)** Percentage (%) of ATP levels after 48 h in the presence of rapamycin. **(F)** Dose-response curve and calculated IC_50_ for adult worm viability, based on the relative motility index, in response to *in vitro* incubation with rapamycin for 48 h, fitted using a five-parameter logistic (5-PL) model. Significance of differences between the rapamycin-treated and the DMSO control groups were assessed by two-way **(A–C)** or one-way ANOVA **(D, E)** with Dunnett’s *post-hoc* correction. Significance: (*) = p <0.05, (**) = p <0.01, (***) = p <0.001, and (****) = p <0.0001. Values are expressed as the mean ± SEM of three to four independent biological replicates.

Incubation with rapamycin at concentrations above 6.25 μM significantly compromised oviposition (**Figure 3D**), indicating that rapamycin might also impair schistosomes reproductive capacity. At 6.25 μM, an 86.5% decrease in egg count was noticed, while concentrations above 25 μM completely ceased oviposition (**Figure 3D**). In addition, eggs laid were smaller, presented poorly developed spicules, and exhibited an abnormal morphology (**Supplementary Figure S2**), indicating a particular impairment in schistosomes reproduction following rapamycin-exposure. Moreover, a severe depletion of total ATP levels of adult parasites was observed following incubation with rapamycin. At 12.5 μM, a 78.7% decrease in ATP levels was noticed (**Figure 3E**). The IC_50_ for rapamycin against adult *S. mansoni* worms, calculated based on worm motility, was 4.6 μM (**Figure 3F**). The calculated IC_50_ values based on pairing, adhesion, egg count, and percentage of ATP are shown in **Supplementary Figure S3**. Positive control worms incubated with PZQ at 1.5 μg/ml was used and data can be found in **Supplementary Figure S4**.

### 3.4 Rapamycin *in vitro* incubation induces modulation in expression of autophagy genes in male and female *S. mansoni* worms

Since rapamycin showed a modulatory potential on the expression levels of autophagy genes in schistosomula (**Figures 2A, B**) and a robust impairment in *S. mansoni* adult worm viability (**Figures 3A–E**), we investigated the possible modulation of autophagy genes induced by rapamycin in adult worms. Besides the five candidates investigated in schistosomula under rapamycin incubation (*Smp_073400.1*, *Smp_301430.1*, *Smp_122910.1*, *Smp_073790.1*, and *Smp_087940.2*), we evaluated key components of the autophagy machinery described in other organisms and for which the orthologue genes were previously identified in *S. mansoni* (Mughal et al., 2021), including *Beclin* (*Smp_048990.1*), *Ambra1* (*Smp_ 156350.1*), *Vps34* (*Smp_128130.1*), *DAP* (*Smp_023840.1*), and *DRAM* (*Smp_006750.2*). Five out of these ten autophagy-associated genes tested were significantly modulated in *S. mansoni* females following incubation with rapamycin, while in male worms, four out of the ten genes were significantly modulated (**Figure 4**).

**Figure 4:**
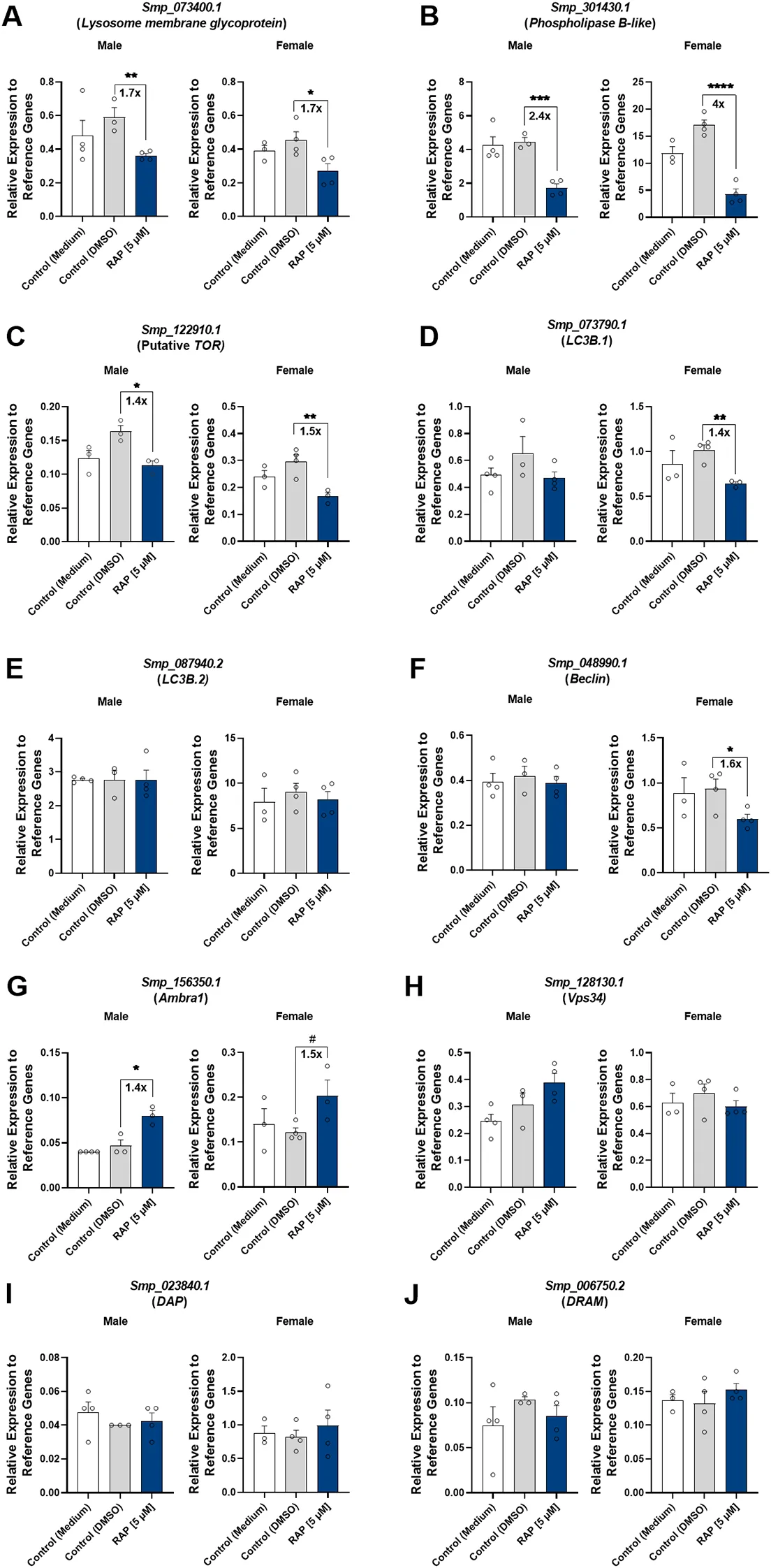
Effect of rapamycin on modulation of the expression levels of autophagy genes in adult male and female *Schistosoma mansoni* worms. Relative transcript levels to reference genes are shown for male (left) and female (right) worms. Relative transcript levels of **(A)** *Lysosome membrane glycoprotein* (*Smp_073400.1*), **(B)** *Phospholipase B-like* (*Smp_301430.1*), **(C)** putative *TOR* (*Smp_122910.1*), **(D)** *LC3B.1* (*Smp_073790.1*), **(E)** *LC3B.2* (*Smp_087940.2*), **(F)** *Beclin* (*Smp_048990.1*), **(G)** *Ambra1* (*Smp_156350.1*), **(H)** *Vps34* (*Smp_128130.1*), **(I)** *DAP* (*Smp_023840.1*), and **(J)** *DRAM* (*Smp_006750.2*), determined by RT-qPCR following *in vitro* incubation of adult worms for 48 h with rapamycin at 5 µM. Expression levels were normalized to the reference genes *Smp_062630.1* and *Smp_128280.1* for males, and *Smp_101310.1* and *Smp_128280.1* for females. Significance of differences between the rapamycin-treated and the DMSO control groups were assessed by unpaired two-tailed Student’s *t*-test, and FCs values are shown below the brackets. Significance levels: (#) = p <0.051, (*) = p <0.05, (**) = p <0.01, (***) = p <0.001, and (****) = p <0.0001. Data are presented as the mean ± SEM of three to four independent biological replicates. RAP: rapamycin.

Similar to the effect observed in schistosomula under rapamycin incubation (**Figures 2A, B**), adult worms also showed a decreased expression of *Smp_073400.1* and *Smp_301430.1.* One late-autophagy gene, related to lysosome biogenesis, showed rapamycin-induced modulation: *Lysosome-associated membrane glycoprotein* (*Smp_073400.1*) was 1.7-fold downregulated by rapamycin in both males and females (**Figure 4A**). In addition, *Phospholipase B-like* (*Smp_301430.1*) was modulated following rapamycin exposure, showing a 2.4- and a 4-fold downregulation in males and females, respectively (**Figure 4B**). Moreover, the putative *TOR* orthologue gene was downregulated in both sexes, showing a 1.4- and 1.5-fold downregulation upon rapamycin incubation (**Figure 4C**) in male and female worms, respectively. The putative *LC3B.1* (*Smp_073790.1)* exhibited a 1.4-fold downregulation in females (**Figure 4D**), while *LC3B.2* (*Smp_087940.2*) was not modulated (**Figure 4E**). *Beclin* (*Smp_048990.1*), one of the most relevant components of the pro-autophagic core complex, was 1.6-fold downregulated following rapamycin exposure in females, but not in males (**Figure 4F**). In contrast, another pro-autophagic core member, *Ambra1* (*Smp_156350.1*), was 1.4- and 1.5-fold upregulated by rapamycin in males and females, respectively (**Figure 4G**). Other autophagy genes, including *Vps34* (*Smp_128130.1*), *DAP* (*Smp_023840.1*), and *DRAM* (*Smp_006750.2*) showed modulation neither in male nor in female worms (**Figures 4H–J**).

### 3.5 Classical autophagy inhibitors do not significantly reduce ATP levels in *S. mansoni* schistosomula

Although the antischistosomal potential of some classical autophagy inhibitors was previously evaluated in *S. mansoni* adult parasites (Mughal et al., 2021; Keiser et al., 2014), their effects on schistosomula viability remain largely unknown. To evaluate the antischistosomal activity of four compounds commonly used to disrupt autophagy against 3–day-old *S. mansoni* schistosomula, parasites were exposed *in vitro* to bafilomycin A1 (0.005 – 2.5 μM), chloroquine (0.4 – 200 μM), wortmannin (0.2 – 100 μM), or spautin-1 (0.4 – 400 μM) at serial two-fold dilutions for up to 120 h, with viability assessed daily by ATP quantification. In contrast to their effect previously shown (Mughal et al., 2021; Keiser et al., 2014) on adult *S. mansoni* parasites, no impairment in schistosomula viability, based on ATP levels, was noticed following incubation with bafilomycin A1 (**Figure 5A**), chloroquine (**Figure 5C**), wortmannin (**Figure 5E**), or spautin-1 (**Figure 5G**).

**Figure 5:**
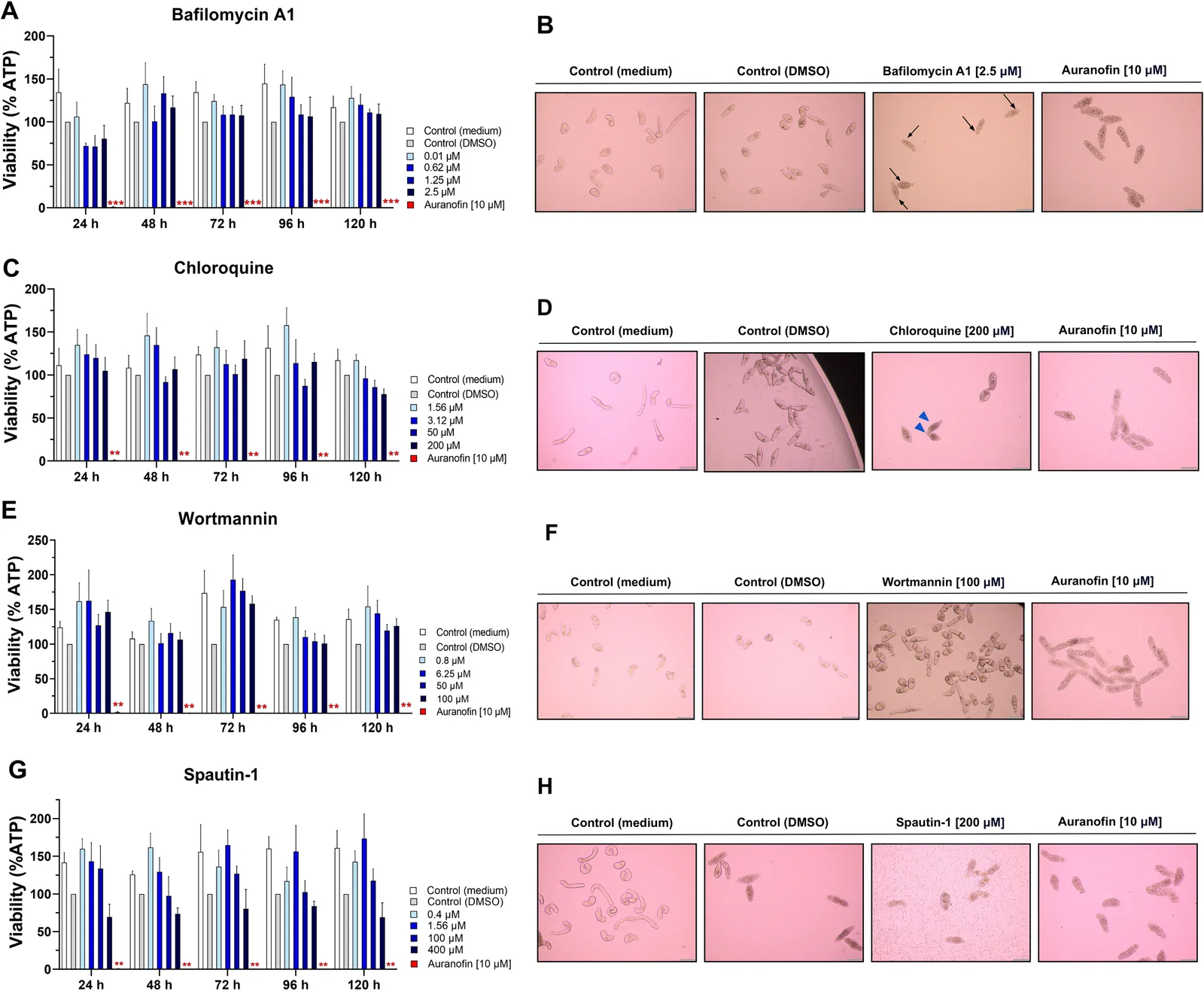
Effect of classical autophagy inhibitors on *Schistosoma mansoni* schistosomula viability. Schistosomula were incubated *in vitro* for up to 120 h with each of the four tested compounds, each at ten different drug concentrations, and parasite viability was monitored daily by assessing their total ATP contents. Four representative concentrations are shown for each compound. **(A)** Bafilomycin A1 effect on schistosomula viability (% ATP). **(B)** Morphological appearance of control schistosomula or parasites incubated with bafilomycin A1 at 2.5 μM for 120 h. **(C)** Chloroquine effect on schistosomula viability (% ATP). **(D)** Morphological appearance of control schistosomula or parasites incubated with chloroquine at 200 μM for 120 h. **(E)** Wortmannin effect on schistosomula viability (% ATP). **(F)** Morphological appearance of control schistosomula or parasites incubated with wortmannin at 100 μM for 120 h. **(G)** Spautin-1 effect on schistosomula viability (% ATP). **(H)** Morphological appearance of control schistosomula or parasites incubated with spautin-1 at 200 μM for 120 h. Black arrows point to the presence of vacuoles; blue triangles point to darker schistosomula. Bar at the bottom right: 100 μm. Auranofin at 10 μM for 120 h was used as positive control. Statistically significant differences were determined by two-way ANOVA with Dunnett’s *post-hoc* correction, comparing each treatment group to the DMSO control group. Significance: (**) = p <0.01, (***) = p <0.001. Data are presented as the mean ± SEM of three to four independent biological replicates.

Despite the absence of impairment in schistosomula ATP levels following *in vitro* exposure to the tested compounds, a few morphological alterations were seen following incubation of parasites with lysosomotropic compounds (bafilomycin A1 or chloroquine), an effect not observed in parasites incubated with spautin-1 or wortmannin. Following exposure to bafilomycin A1, schistosomula showed the presence of vacuoles and a darker appearance (**Figure 5B**). In addition, incubation for 120 h with bafilomycin A1 at concentrations above 0.31 μM led to a complete absence of movements on parasites (**Supplementary Video 4**). Similarly, following exposure to chloroquine, schistosomula appeared darker and swollen (**Figure 5D**), as well as lethargic at concentrations above 50 μM (**Supplementary Video 5**). On the other hand, schistosomula incubated with spautin-1 or wortmannin showed no visible morphological alterations (**Figure 5F, H**, respectively) nor changes in motility (**Supplementary Videos 6**, **7**, respectively). Effect of the positive control auranofin (10 μM) on schistosomula is shown in **Supplementary Video 8**.

### 3.6 Chloroquine triggers modulation in expression levels of autophagy genes in *S. mansoni* schistosomula

Although chloroquine showed no significant reduction in schistosomula ATP levels (**Figure 5C**), the morphological alterations induced by the compound may indicate some level of viability impairment. Therefore, we investigated the effect of chloroquine *in vitro* incubation at 100 μM on late-autophagy genes in schistosomula, including *Lysosome-associated membrane glycoprotein* (*Smp_073400.1*), *Phospholipase B-like* (*Smp_301430.1*), and the two putative *LC3B* (*Smp_073790.1* and *Smp_087940.2*). Interestingly, incubation of schistosomula with chloroquine showed a similar pattern of expression changes on autophagy genes in comparison with the changes under incubation with the classical autophagy inducer rapamycin. Following chloroquine incubation, *Lysosome-associated membrane glycoprotein* (*Smp_073400.1*) (**Figure 6A**) and *Phospholipase B-like* (*Smp_301430.1*) (**Figure 6B**) were downregulated, showing a 2.1- and a 3.1-fold change, respectively, a somewhat less pronounced downregulation compared with that induced by rapamycin incubation (**Figures 2A, B**). The *LC3B* putative orthologue genes (*Smp_073790.1* and *Smp_087940.2*) showed no modulation upon chloroquine incubation (**Figures 6C, D**).

**Figure 6:**
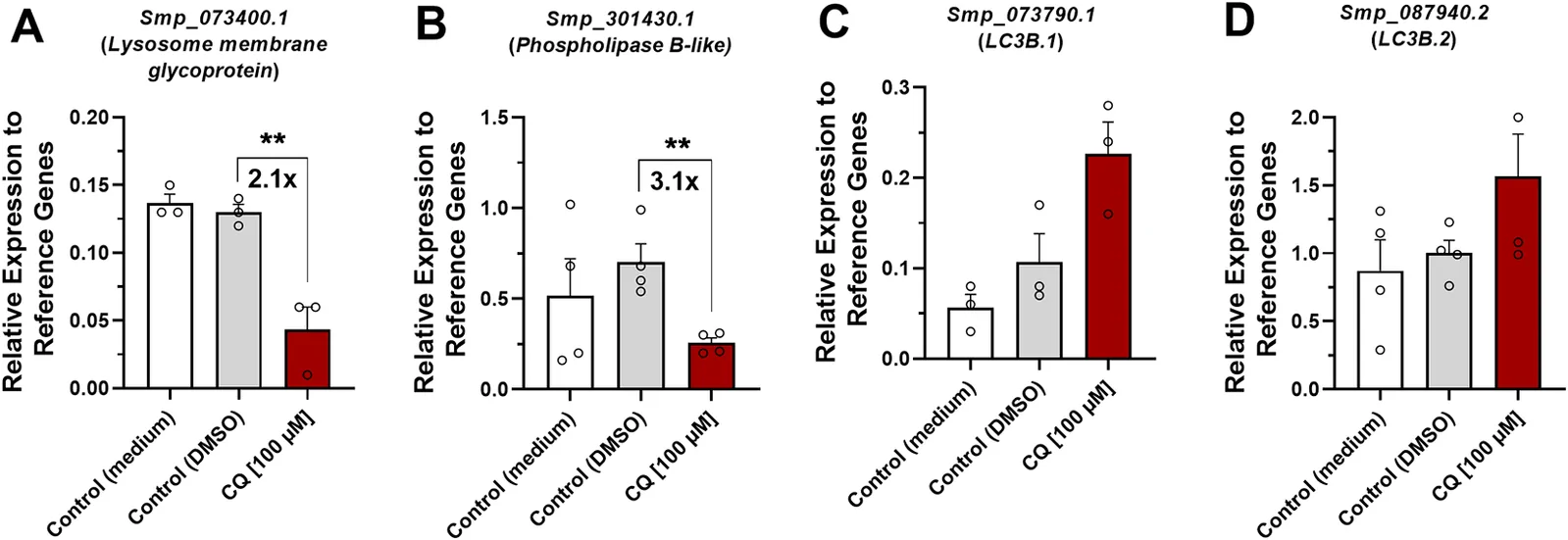
Effect of chloroquine on the expression levels of autophagy genes in *Schistosoma mansoni* schistosomula. Relative transcript levels of **(A)** *Lysosome membrane glycoprotein* (*Smp_073400.1*), **(B)** *Phospholipase B*-*like* (*Smp_301430.1*), **(C)** *LC3B.1* (*Smp_073790.1*), and **(D)** *LC3B.2 (Smp_087940.2*) were determined by RT-qPCR following incubation *in vitro* with chloroquine at 100 μM for 48 h. The relative expression levels were calculated using the delta Ct approach considering each pair of primers efficiency and normalized to the geometric average of two selected reference genes (*Smp_062630.1* and *Smp_041060.1*). Statistically significant differences between the treatment and the DMSO control groups were assessed using unpaired two-tailed Student’s *t*-test, and FC values are shown below the brackets. Significance: (*) = p <0.05, and (**) = p <0.01. Values are expressed as the mean ± SEM of three to five independent biological replicates. CQ, Chloroquine.

### 3.7 Canonical autophagy inhibitors have little impact on the expression levels of autophagy genes in adult *S. mansoni* worms

To investigate the modulatory potential of spautin-1 and chloroquine on autophagy genes in adult *S. mansoni*, worms were incubated *in vitro* with 50 μM spautin-1 or with 200 μM chloroquine for 48 h. Phenotypic parameters following incubation with spautin-1, previously characterized in the *S. mansoni* Liberian strain (Mughal et al., 2021), were recorded here for the *S. mansoni* BH strain. When replicating in the BH strain the experimental conditions described by (Mughal et al., 2021), we observed a higher sensitivity of the BH strain to spautin-1 compared with the Liberian strain used in the previous study (Mughal et al., 2021) (**Figure 7**). Pairing and adhesion were completely ceased after 48 h of incubation with spautin-1 (**Figures 7A, B**), in contrast to approximately 25% decrease in worm pairing observed in the Liberian strain following incubation with spautin-1 at the same concentration (Mughal et al., 2021). In addition, effects on motility were also more prominent on the BH strain compared with the Liberian strain. On the BH strain, a 91% decrease in motility was observed after a 48-h incubation (**Figure 7C**), whereas a significant decrease in the motility on the Liberian strain at 50 μM was observed only after 96 h of drug exposure (Mughal et al., 2021).

**Figure 7:**
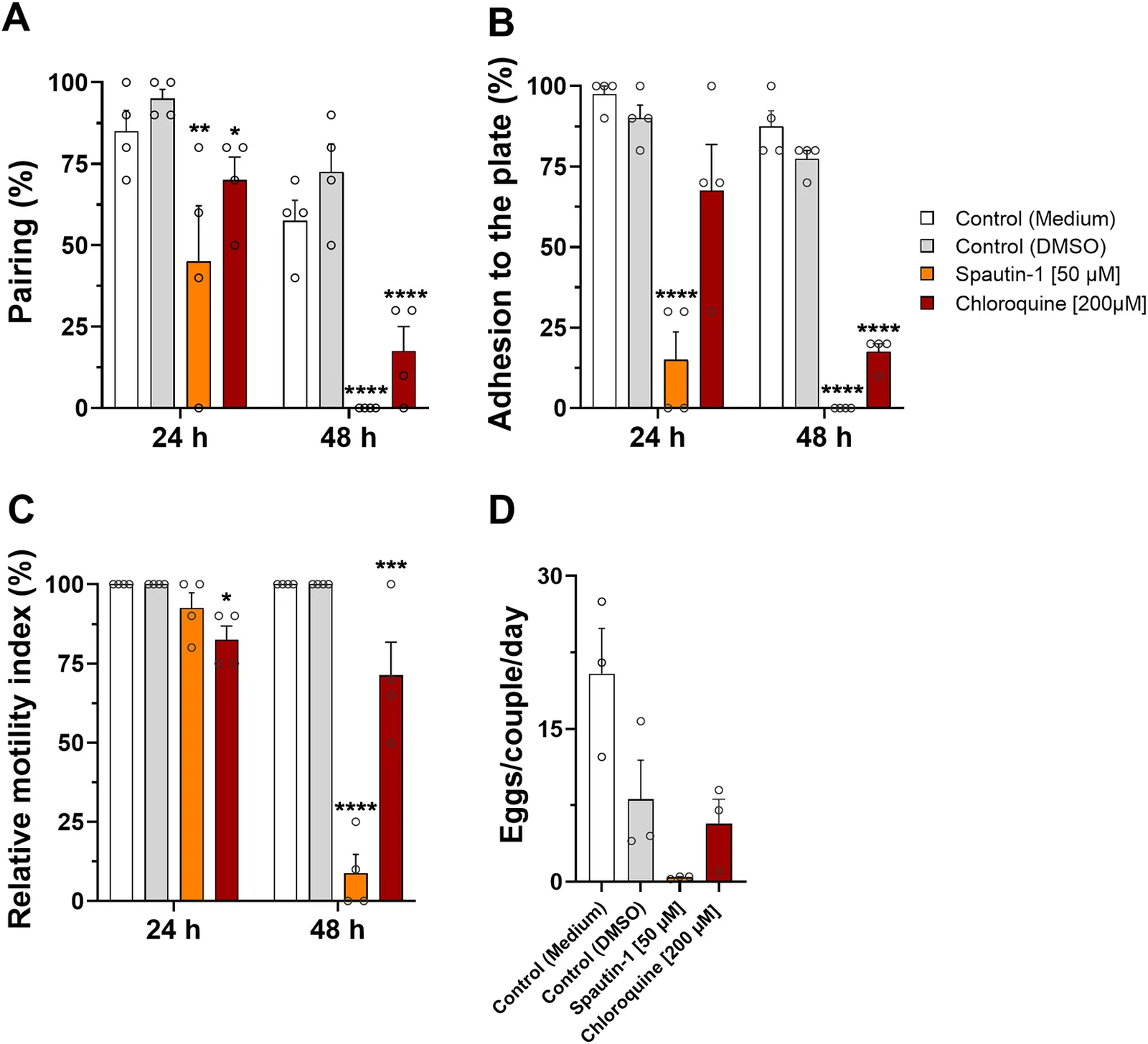
Evaluation of adult *S. mansoni* (BH strain) worm viability following *in vitro* incubation with spautin-1 or chloroquine. Spautin-1 at 50 μM (orange) or chloroquine at 200 μM (red) were added to adult couples *in vitro* and the phenotypic parameters were recorded after 24 or 48 h incubation, as indicated on the x-axis. As negative control, DMSO (0.1% v/v) was used. **(A)** Percentage (%) of paired worms. **(B)** Percentage (%) of couples attached to the plate. **(C)** Relative motility index of couples. **(D)** Number of eggs laid per couple per day. The significance of differences between treated and control (DMSO) worms were assessed by two-way **(A–C)** or one-way ANOVA **(D)** with Dunnett’s *post-hoc* correction. Significance: (*) = p <0.05, (**) = p <0.01, (***) = p <0.001, and (****) = p <0.0001. Values are expressed as mean ± SEM of three to four independent biological replicates.

Similarly, chloroquine incubation showed a significant decrease in worm pairing, adhesion, and motility (**Figures 7A–C**). Additionally, a decrease in egg count was observed, although not statistically significant (**Figure 7D**), along with impaired eggs laid by adult worms incubated with chloroquine, which appeared poorly developed (**Supplementary Figure S5**).

Subsequently, we investigated the impact of spautin-1 or chloroquine on the expression of autophagy genes in adult *S. mansoni* worms and observed minimal effect on gene expression modulation (**Figures 8A–I**). Thus, despite the previously reported effect of 50 μM spautin-1 on worm viability (**Figure 7**), no modulation on the transcript levels of the autophagy-associated genes tested was observed (**Figure 8**). Similarly, incubation with chloroquine showed little impact on the selected autophagy genes, with only one out of nine tested genes, *Phospholipase B-like* (*Smp_301430.1)*, showing a 2-fold downregulation in female worms (**Figures 8A–I**).

**Figure 8:**
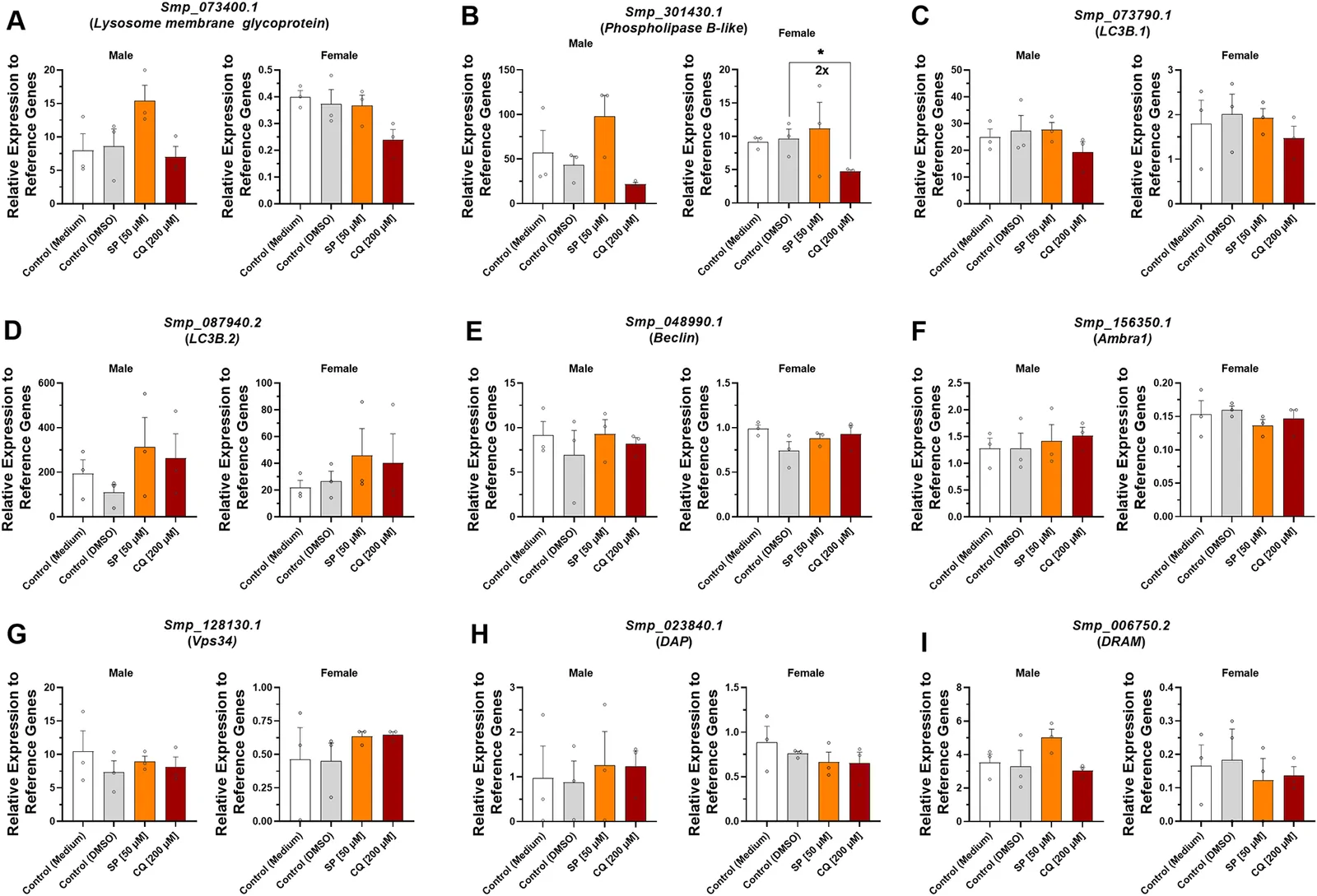
Effect of spautin-1 or chloroquine on the expression of autophagy genes in adult male and female *S. mansoni* worms. Relative transcript levels to reference genes are shown for male (left) and female (right) worms. Relative transcript levels of **(A)** *Lysosome membrane glycoprotein* (*Smp_073400.1*), **(B)** *Phospholipase B-like* (*Smp_301430.1*), **(C)** *LC3B.1* (*Smp_073790.1*), **(D)** *LC3B.2* (*Smp_087940.2*), **(E)** *Beclin* (*Smp_048990.1*), **(F)** *Ambra1* (*Smp_156350.1*), **(G)** *Vps34* (*Smp_128130.1*), **(H)** *DAP* (Smp_023840.1), and **(I)** *DRAM* (Smp_006750.2), determined by RT-qPCR following *in vitro* incubation with spautin-1 (50 μM) or chloroquine (200 μM) for 48 h. For male worms, expression levels were normalized to the reference genes *Smp_341650.1* and *Smp_101310.1*, while for females, *Smp_101310.1* and *Smp_128280.1* were used. Significance of differences between treated and DMSO control groups were assessed by unpaired two-tailed Student’s *t*-test. Significance: (*) = p <0.05. Data are presented as the mean ± SEM of three independent biological replicates. SP, Spautin-1; CQ, Chloroquine.

## 4 Discussion

In this study, we characterized *S. mansoni* adult worms and schistosomula viability upon incubation with compounds known to modulate autophagy in other organisms, since this pathway remains poorly understood in schistosomes. In addition to evaluating the *in vitro* phenotypic effects of rapamycin on both adult worms and schistosomula, we described a modulation of the expression levels of autophagy genes induced by this drug. Furthermore, we screened, against *S. mansoni* schistosomula, four compounds known to inhibit autophagy in many organisms and characterized changes in gene expression induced by chloroquine. These results highlight the antischistosomal potential of classical autophagy modulators as key pharmacological tools for investigating this pathway. Moreover, our findings reveal the capacity of classical autophagy modulators to trigger gene expression modulation in *S. mansoni*, a phenomenon still unexplored in the field, as autophagy regulation is usually primarily evaluated at the post-translational level.

Autophagy modulators represent attractive candidates for investigating autophagic pathways (Bu et al., 2022; Kench et al., 2024). Moreover, clinically approved compounds have emerged as a rapid and cost-effective strategy in drug development (Pushpakom et al., 2019), which is particularly relevant in the context of parasitic diseases, a historically underfunded area (Weng et al., 2018). First, our findings demonstrated that both intra-mammalian *S. mansoni* stages are sensitive to rapamycin, with adult worms exhibiting a particularly higher sensitivity compared with schistosomula, as evidenced by rapamycin 3.2-fold lower IC_50_ value in adult stages. In contrast to the findings reported by (Rossi et al., 2002), a severe impairment in *S. mansoni* adult worm viability was observed, including reduced worm pairing, adhesion, motility, and ATP levels following a 48-h incubation. Reproductive capacity was also compromised, with treated schistosomes laying fewer and poorly developed eggs. In schistosomula, ATP levels were drastically reduced at concentrations above 25 μM, with mortality confirmed by PI/FDA staining, alongside marked morphological and motility alterations. These phenotypic effects observed following rapamycin exposure are consistent with previous reports of the anthelmintic potential of rapamycin against *Echinococcus granulosus* (Cumino et al., 2010), and other autophagy-inducing compounds against *S. mansoni*, including metformin (Abdel Menaem et al., 2025) and imatinib (Mughal et al., 2021). These effects might be attributed to the upregulation of the autophagy pathway, since rapamycin autophagy-modulatory potential has been previously shown to increase the key autophagy marker LC3B in *S. mansoni* adult worms (Mughal et al., 2021). However, further validation using direct measurements of autophagic flux in schistosomula is indeed needed to confirm this hypothesis, since autophagy is predominantly regulated at the post-translational level. Taken together, these results emphasize the underexplored potential of rapamycin as a relevant compound to be further explored against *S. mansoni*.

In addition, it is worth noting that both rapamycin and chloroquine are FDA-approved, clinically available drugs. It is important to consider that rapamycin’s IC_50_ values may initially appear to challenge its translational potential, as rapamycin is typically used clinically within a narrow therapeutic window of 5–15 ng/ml (MacDonald et al., 2000), a concentration considerably lower than the IC_50_ values obtained in our assays. Nevertheless, several rapalogs are already clinically available and exhibit improved pharmacological properties, representing potential chemical probes for investigating schistosomes biology in future studies.

Changes in gene expression in other organisms driven by rapamycin have been described in different contexts (Dai et al., 2012; Raynes et al., 2024). In this work, we described the impact of rapamycin on the expression of a putative *TOR* orthologue gene (*Smp_122910.1*), which was significantly downregulated in male and female worms upon rapamycin incubation, whereas no significant modulation was observed in schistosomula. In most eukaryotes, the *mTORC1* complex is allosterically inhibited by rapamycin (Sabers et al., 1995); this master regulator governs the autophagy-initiating ULK complex and the phosphorylation of various ATGs (Lee et al., 2022). While the host *mTOR* pathway is known to respond to parasitic infections (Rashidi et al., 2021), accumulating evidence suggests that *TOR* signaling may also operate within the parasites themselves (Barquilla et al., 2008). Interestingly, *Smp_122910.1* is not differentially expressed in *S. mansoni* under other stress conditions, such as incubation with 5-azacytidine (Geyer et al., 2018) and *in vivo* administration of PZQ (McCusker et al., 2021), or under conditions that are known to modulate the late autophagy pathway in schistosomes, including incubation with self-cured rhesus macaque plasma (Amaral et al., 2021) or with imatinib (Buro et al., 2014). This suggests that the *S. mansoni TOR* is not modulated by stress conditions or general conditions that appear to disturb autophagy but rather represents a specific rapamycin-induced response. Although the *TOR* expression levels appear to be similar across stages – 13.5, 8.4, and 11.5 TPM (transcripts per million) in *S. mansoni* males, females, and schistosomula, respectively – its single-cell RNA-Seq spatial expression differs remarkably. In adult worms, the putative *TOR* orthologue gene is broadly expressed (**Supplementary Figure S6C**), whereas in schistosomula, *TOR* expression is enriched in discrete cell clusters, yet overall spatial expression is considerably reduced compared with adult stages (**Supplementary Figure S7C**). This ubiquitous distribution in adults may account for their higher sensitivity to rapamycin, as broadly expressed genes often play essential roles in maintaining cellular homeostasis and survival, whereas tissue-specific genes are typically involved in specialized biological functions (Gu et al., 2023). This transcriptional modulation of *TOR* is comparatively less described, as *mTOR* regulation is primarily documented at the post-translational level, focusing on the phosphorylation of downstream autophagy components (Marques-Ramos and Cervantes, 2023). Nevertheless, transcriptional (Dai et al., 2012; Riquelme et al., 2016) and translational (Vilà et al., 2012; Rogers-Broadway et al., 2019) regulation of *mTOR* itself has been observed under different conditions, leading to an emerging interest in its genetic control (Audet-Walsh et al., 2020; Marques-Ramos and Cervantes, 2023). Indeed, our findings in *S. mansoni* demonstrate a significant response of the putative *TOR* gene to rapamycin at the gene expression level in adult worms. However, whether this modest downregulation exerts a biologically meaningful role in schistosomes remains to be further explored, for instance through functional approaches, as does the precise link between *TOR* transcriptional modulation and the classical post-translational modulation via phosphorylation.

We have demonstrated that classical drugs known to inhibit autophagy in many other organisms, including bafilomycin A1, spautin-1, and wortmannin, had little impact on schistosomula ATP levels. These compounds are known to exhibit *in vitro* antischistosomal properties against adult worms (Mughal et al., 2021). Chloroquine, which was previously reported to have no effect on NTS at 10 and 33 μM (Keiser et al., 2014), also showed no significant impact on the ATP stock levels of 3-day-old schistosomula, even at a concentration as high as 200 μM. However, in contrast to schistosomula, adult worms were sensitive to chloroquine at 200 μM, showing impaired pairing, motility, and egg quality. Furthermore, we found here the BH strain to be more sensitive to spautin-1 than the Liberian strain (Mughal et al., 2021). Notably, differential responses to drug exposure have been previously reported among *S. mansoni* strains (Valentini et al., 2025), which are likely due to genetic variability affecting drug sensitivity, and therefore, this warrants further investigation.

Notably, while the downregulation of the late autophagy pathway has been related to reduced schistosomula viability (Amaral et al., 2021), we observed that even in the absence of ATP depletion, the two lysosomotropic modulators (bafilomycin A1 and chloroquine) did induce morphological alterations and changes in schistosomula motility. These results suggest a possible impairment of schistosomula viability following drug-exposure through ATP-independent mechanisms, an effect previously observed in other scenarios, such as adult worms following lethal PZQ-exposure, in which worms die without complete depletion of ATP stock levels, as observed in PZQ-treated worms used as a lethal control condition in the work of (Zenga-Carrenho et al., 2026). In this direction, bafilomycin A1 and chloroquine properties against schistosomula should be further investigated using alternative methodologies, including high-resolution microscopy to evaluate tegumental damage, and long-term viability assessments to determine if these morphological changes translate into irreversible parasite death over time.

Importantly, these classical compounds commonly used to disturb autophagy may also exhibit alternative effects unrelated to autophagy. Rapamycin, for instance, regulates several TOR-dependent processes beyond autophagy that may account for the phenotypic changes observed in schistosomula and adult worms, in addition to the transcriptional reprogramming observed in this work. Similarly, some autophagy inhibitors might present pleiotropic effects as well. While chloroquine may demonstrate immunomodulatory effects (Cooper and Magwere, 2008), wortmannin can inhibit all three classes of PI3K, thereby impacting cell growth, cytoskeletal remodeling, and intracellular tracking (Koyasu, 2003). Thus, genetic validation approaches, including RNAi, may be necessary to further investigate the role of autophagy genes in schistosome survival. Moreover, direct evidence of autophagy inhibition induced by these compounds in schistosomula remains to be explored.

Interestingly, under the conditions tested, we observed comparatively little effect of the canonical autophagy inhibitors on schistosomula in parallel to a strong decrease in adult worm viability, which was also observed in the work of (Mughal et al., 2021). Stage-specific effects of autophagy modulators have been described in other parasites, such as *Plasmodium* (Mishra et al., 2025) and *Trypanosoma brucei* (Proto et al., 2014). Assuming that these compounds do modulate autophagy in schistosomes, autophagy primary role in adult worms may be on homeostasis maintenance, such as degradation of unfolded proteins and cellular-components recycling, which aligns with the broadly spatial expression of autophagy genes observed in adult single-cell RNA-Seq data (**Supplementary Figure S6**). Consequently, targeting autophagy imposes a severe physiological stress leading to impairment in viability and reproduction, as observed here and by (Mughal et al., 2021). Conversely, autophagy is also closely linked to development (Hale et al., 2013), particularly in parasites (Walker et al., 2013; Vanrell et al., 2017). In *Plasmodium*, for example, ATG7 was found to be essential for the development of *Plasmodium berghei* blood and liver stages, but not sporozoites (Mishra et al., 2025). In schistosomes, autophagy has been implicated in maturation, with the downregulation of late-autophagy genes hindering worm development (Amaral et al., 2021). Curiously, half of the genes tested in the present work, including *Ambra1* (*Smp_156350.1*), *Vps34* (*Smp_128130.1*), *DRAM* (*Smp_006750.2*), *Phospholipase B-like* (*Smp_301430.1*), and *TOR* (*Smp_122910.1*), exhibit spatially discrete expression in schistosomula single-cell RNA-Seq data (**Supplementary Figure S7**), but become broadly expressed across multiple tissues in adult worms (**Supplementary Figure S6**). This suggests a progressive expansion of the autophagic transcriptional program throughout the parasite’s life cycle, with broader expression in adult worms. The molecular mechanisms underlying these stage-specific autophagic properties remain unclear and warrant further investigation to fully elucidate the role of autophagy machinery across *S. mansoni* life-cycle stages.

Autophagy genes play a pivotal role in parasites other than *S. mansoni*, regulating lifespan (Powers et al., 2006), homeostasis (Cull et al., 2014), and development (Proto et al., 2014), underscoring the relevance of investigating these genes in schistosomes as well. In this study, we observed that compounds commonly used to disturb autophagy in other organisms showed a mostly modest modulation on the expression levels of canonical autophagy genes, including members of the pro-autophagic core complex regulating the autophagosome biogenesis (He and Levine, 2010), such as *Beclin* and *Ambra1*. In contrast, the expression levels of other well-characterized autophagy mediators, namely *Vps34* and *DRAM*, remained unaffected upon incubation of *S. mansoni* with autophagy modulators. This finding aligns with reports that starvation, a canonical autophagy-inducing condition, similarly fails to alter the expression levels of these components of the autophagy machinery (Mughal et al., 2021). Furthermore, *Smp_087940.2* (*LC3B.2*) remained unmodulated across all conditions tested, while *LC3B.1* (*Smp_073790.1*) was significantly modulated in female worms following rapamycin incubation and in schistosomula after chloroquine treatment. The lack of gene expression modulation for these markers in other conditions does not preclude alterations at the protein level, since *LC3* activation is primarily regulated post-translationally through its lipidation. While this has been previously reported at the protein level in *S. mansoni* adult worms (Mughal et al., 2021), our data suggest that a similar response may occur in schistosomula through post-transcriptional or post-translational mechanisms, although this hypothesis remains to be experimentally confirmed. Collectively, these observations highlight the complexity of autophagy regulation in *S. mansoni* and reinforce the need for complementary post-translational analyses to fully characterize the functional role of these conserved regulators in this parasite. Importantly, the modulation of transcript levels in adult worms must be interpreted with caution, as the transcriptional responses cannot be uniquely attributed to drug action. They may instead reflect secondary responses to severe toxicity, stress, altered reproductive physiology, or unpairing; such effects are especially pronounced in females, given their profound, pairing-dependent transcriptional activity and reproductive physiology.

Notably, *Smp_301430.1* and *Smp_073400.1* were significantly downregulated under incubation with rapamycin in schistosomula, adult male and adult female worms; while also significantly downregulated under incubation with chloroquine in schistosomula and female worms. Additionally, *Smp_301430.1* showed an upregulation in males under spautin-1 incubation. *Smp_301430.1* codes for a *Phospholipase B-like* protein; phospholipases have been linked to autophagy in various contexts, including plant physiology (Yao et al., 2022; Guan et al., 2025), arthritis (Fu et al., 2023), and cancer (Hwang et al., 2014; Shimozawa et al., 2017). *Smp_073400.1* codes for a putative *Lysosome-associated membrane glycoprotein*, a well-expressed component in *S. mansoni* (Al-Adhami et al., 2005). This protein is annotated in KEGG pathways as component of the major lysosomal membrane proteins (LAMPs), a group exhibiting a crucial role in limiting the potent activities of lysosomal acid hydrolases to the interior of lysosomes (Eskelinen, 2006) and facilitating their fusion with other organelles (Huynh et al., 2007). It is intriguing that *Smp_073400.1*, a gene involved in lysosome integrity, was downregulated following exposure to rapamycin (a canonical autophagy inducer) and chloroquine (a canonical autophagy inhibitor). Since the expression of *LAMPs* on the cell surface has been postulated to protect the plasma membrane from degradation by lysosomal hydrolases (Fukuda, 1991), its downregulation could compromise cellular integrity, leading to physiological stress and impaired viability. These two genes (*Smp_073400.1* and *Smp_301430.1*) are highly expressed in *S. mansoni* adult worms, but comparatively less expressed in schistosomula. For instance, *Smp_073400.1* is greatly expressed in adult worms (1,325 and 355 TPM in male and females, respectively) but shows an approximately 200 TPM expression in schistosomula (**Supplementary Figure S8A**). Similarly, *Smp_301430.1* shows an average 193 and 297 TPM in male and females, respectively, and approximately 30 TPM in schistosomula (**Supplementary Figure S8B**). In adult worms, both genes are ubiquitously expressed, with a particularly notable enrichment on the parenchyma cluster (**Supplementary Figure S6**), similarly to the upregulation of autophagy gene *DAP* in starved planarians (González-Estévez et al., 2007). Consistent with our findings, these two genes were previously reported to be downregulated in *S. mansoni* under the immune-stress conditions alongside other late-autophagy genes (Amaral et al., 2021), and following incubation with 5-azacytidine, a methyltransferase inhibitor known to modulate autophagy in other models (Marycz et al., 2019; Filip et al., 2022), further suggesting they may belong to a core stress-responsive autophagic signature in *S. mansoni*.

Overall, we have characterized the antischistosomal potential of rapamycin against two intra-mammalian stages of *S. mansoni*. Crucially, a key novelty of this work lies in the characterization of autophagy genes modulated by rapamycin, indicating the putative *TOR* orthologue (*Smp_122910.1*) and other two drug-responsive candidate genes (*Smp_073400.1* and *Smp_301430.1*) as promising targets for further functional assays, as well as investigation of direct TOR signaling and autophagy flux in schistosomes. Moreover, we have described that compounds commonly used to perturb autophagy produce distinct phenotypic and transcriptional responses in *S. mansoni* adult worms and schistosomula. To our knowledge, this represents the first transcript-level analysis under incubation with canonical autophagy modulators in schistosomes, bridging a critical gap in the understanding of schistosome biology. Future studies should expand upon these findings by integrating complementary approaches, including large-scale transcriptomic and proteomic analyses under autophagy modulation, RNAi-mediated silencing of key regulators, and gene/protein interaction network analyses, which may reveal broader regulatory hubs within autophagy genes and accelerate the identification of potential druggable targets. Collectively, the present findings highlight compounds commonly used to disturb autophagy as promising pharmacological tools that can impair schistosomes viability and induce transcriptional changes. This underscores their potential as putative frameworks to possibly unravel new targets to tackle schistosomiasis.

## Data Availability

The original contributions presented in the study are included in the article/**Supplementary Material**. Further inquiries can be directed to the corresponding author.
