# Supplementary material for: Classical autophagy-modulating compounds impair viability and alter autophagy gene expression in schistosomula and adult Schistosoma mansoni
Source: Front Parasitol. 2026 Oct 27;5:1892940. doi: 10.3389/fpara.2026.1892940 (PMC13748313; doi:10.3389/fpara.2026.1892940)
Supplement: Supplementary file 1 [file SupplementaryFile1.docx]

**Supplementary Figure S1: Phenotypic appearance of *Schistosoma mansoni* schistosomula following incubation with rapamycin.** Images show schistosomula incubated *in vitro* with rapamycin at 25 or 50 μM for 96 h. All schistosomula incubated with rapamycin appeared dark, swollen and with a granular appearance. Negative controls shown include medium alone and vehicle (DMSO at 12 % v/v), and positive control auranofin at 10 μM. Black arrows point to the presence of vacuoles. Grey bar at the bottom right: 100 μm.

**Supplementary Figure S2: Morphological alterations in *S. mansoni* eggs induced by incubation with rapamycin.** Adult *S. mansoni* worms incubated *in vitro* with rapamycin at 5 μM for 48 h showed impaired oviposition. Panels show eggs laid *in vitro* by **(A)** control group (medium), **(B)** control group (DMSO at 0.05% v/v), and **(C)** rapamycin-treated group. Black arrows indicate poorly developed eggs. Grey bar at the bottom right: 100 μm.

**Supplementary Figure S3: Dose-response curves based on different phenotypic parameters following incubation of adult *S. mansoni* worms with rapamycin.** Adult *S. mansoni* were *in vitro* incubated with rapamycin for 48 h, and relative viability was assessed in comparison to the control group (DMSO at 0.05% v/v). Dose-response curves and the respective IC_50_ values for adult worm viability were determined based on **(A)** pairing, **(B)** adhesion to the plates, **(C)** oviposition, and **(D)** viability measured by ATP levels, fitted using a five-parameter logistic (5-PL) model. Data are presented as mean ± SEM of three to four independent biological replicates.

**Supplementary Figure S4: Phenotypic effects on adult *S. mansoni* worms of praziquantel (PZQ), used as a positive control.** Adult *S. mansoni* worms were incubated *in vitro* with PZQ at 1.5 µg/ml for 48 h and compared with a control group (DMSO at 0.05 % v/v). **(A)** Percentage (%) of paired worms. **(B)** Percentage (%) of couples attached to the plate. **(C)** Relative motility index. **(D)** Number of eggs laid per couple per day. **(E)** Quantification (%) of ATP levels. The significance of differences between PZQ-exposed and control (DMSO) worms was assessed by two-way **(A-C)** or one-way ANOVA **(D, E)** with Dunnetts’ post-hoc correction. Significance: (**) = p <0.01, (***) = p <0.001, and (****) = p <0.0001. Data are presented as mean ± SEM of two independent biological replicates.

**Supplementary Figure S5: Morphological alterations in *S. mansoni* eggs induced by incubation with chloroquine.** Adult *S. mansoni* worms incubated *in vitro* with chloroquine at 200 μM for 48 h showed impaired oviposition. Panels show eggs laid *in vitro* by **(A)** control group (medium), **(B)** control group (DMSO at 0.1% v/v), and **(C)** chloroquine-treated group. Black arrows indicate poorly developed eggs. Grey bar at the bottom right: 100 μm.

**Supplementary Figure S6: Single-cell cluster expression profiles on adult *S. mansoni* worms of the ten autophagy genes, selected for RT-qPCR quantification following *in vitro* exposure to the tested compounds.** Blue circles highlight particular enrichment of the parenchyma cluster. UMAP plots show the expression enrichment of genes and are colored by gene expression (white = low, red = high). The scale represents log_10_ (UMIs+1). Single-cell expression data is available online at <https://schisto.xyz/>.

**Supplementary Figure S7: Single-cell cluster expression profiles on *S. mansoni* schistosomula of the ten autophagy genes, selected for RT-qPCR quantification following *in vitro* exposure to the tested compounds.** UMAP plots show the expression enrichment of genes and are colored by gene expression (white = low, red = high). The scale represents log_10_ (UMIs+1). Single-cell expression data is available online at <https://schisto.xyz/>.

**Supplementary Figure S8: Expression levels (expressed in TPM – transcripts per million) of ten selected autophagy genes across six life-cycle stages of *S. mansoni*, selected for RT-qPCR quantification following *in vitro* exposure to the tested compounds.** Gene identities are indicated at the top of each panel. Data available at https://verjolab.shinyapps.io/Reference-genes. M/S = Miracidia/Sporocysts. C = Cercariae. So = Schistosomula. M = adult Males. F = adult Females.

**Supplementary Videos:**

Supplementary video 1: Control (medium) schistosomula incubated *in vitro* for 120 h.

Supplementary video 2: Control (DMSO) schistosomula incubated *in vitro* for 120 h.

Supplementary video 3: Schistosomula incubated *in vitro* with rapamycin at 25 μM for 120 h.

Supplementary video 4: Schistosomula incubated *in vitro* with bafilomycin A1 at 0.31 μM for 120 h.

Supplementary video 5: Schistosomula incubated *in vitro* with chloroquine at 50 μM for 120 h.

Supplementary video 6: Schistosomula incubated *in vitro* with wortmannin at 100 μM for 120 h.

Supplementary video 7: Schistosomula incubated *in vitro* with spautin-1 at 100 μM for 120 h.

Supplementary video 8: Schistosomula incubated *in vitro* with auranofin at 10 μM (positive control) for 120 h.
